# Increasing Age Is Associated with Worse Prognostic Factors and Increased Distant Recurrences despite Fewer Sentinel Lymph Node Positives in Melanoma

**DOI:** 10.1155/2012/456987

**Published:** 2012-03-18

**Authors:** A. J. Page, A. Li, A. Hestley, D. Murray, G. W. Carlson, K. A. Delman

**Affiliations:** ^1^Department of Surgery, Emory University School of Medicine, Emory University Hospital, H120, 1364 Clifton Road, Atlanta, GA 30322, USA; ^2^Department of Surgery, Emory University School of Medicine, Emory University and Winship Cancer Institute, Atlanta, GA 30322, USA

## Abstract

*Background*. Advanced age is associated with a poorer prognosis in patients with melanoma. Despite this established finding, a decreased incidence of positive sentinel lymph nodes (SLNs) with advancing age has paradoxically been described. *Methods*. Using a single-institution database of melanoma patients between 1994 and 2009, the relationship between standard clinicopathologic variables and recurrence based on age was evaluated. *Results*. 1244 patients who underwent successful SLN biopsies were analyzed (mean followup 80.3 months). Increasing age was independently associated with worse survival on multivariable analysis (*P* = 0.02). SLN status was more likely to be negative if the patient was older (*P* = 0.01). *Conclusions*. Our data supports the paradox that increasing age is associated with a lower frequency of positive-SLN biopsies despite age itself being a poor prognostic factor. We propose that age-dependent variations in the primary tumor and the patient may predispose to a hematogenous route of spread for the older population, leading to worse survival.

## 1. Introduction

 One of the most significant advances in the management of melanoma has been the adoption of the sentinel lymph node (SLN) technique. This procedure, through the cooperation between surgery, nuclear medicine, and pathology, has enabled surgeons to identify patients with subclinical nodal metastases. For those patients with a positive SLN, more aggressive treatments like lymphadenectomies can be performed, and for those patients with a negative SLN, these potentially morbid procedures may be safely avoided [1].

 For patients with stages I and II melanoma, a positive SLN is the single most important factor that determines overall prognosis and survival [1, 2]. Like SLN status, advanced age is also associated with poorer prognosis in patients with melanoma [1, 3, 4]. Furthermore, previous studies have demonstrated that increasing age is correlated to other poor prognostic features [5]. Paradoxically, in spite of these findings, a decreased incidence of positive sentinel lymph nodes (SLNs) with advancing age has been described [2, 6]. Possible explanations for this observation include age-dependent variations in metastatic spread, with older patients predisposed to distant metastasis via a hematogenous route compared with younger counterparts. We examined this hypothesis by reviewing the recurrence patterns and other clinicopathological variables as a function of age.

## 2. Methods

### 2.1. Patients and Methods

 After obtaining appropriate Emory University Institutional Review Board (IRB) approval, we undertook a retrospective query of our institutional melanoma database all patients undergoing SLN from 1994 to 2009, identifying 1244 patients with complete records. Mean followup was 80.3 months. Pure desmoplastic cases and those without complete data were excluded. Clinical and tumor variables examined included initial SLN status, gender, ulceration, depth, primary tumor site, and age. Recurrence patterns were recorded as local, in situ, regional, or distant.

### 2.2. SLN Mapping and Biopsy

SLN biopsy was performed as described previously [1]. All patients underwent lymphoscintigraphy preoperatively using filtered technetium—99 m—sulfur colloid. Vital blue dye injected at the time of surgery was used routinely in the latter half of the study. Measurements of radioactivity in the radiolabeled lymph nodes were made intraoperatively with a hand-held gamma probe. Peak counts or counts accumulated over a 10-second count were recorded. SLNs were removed until the lymphatic bed count fell to 10 percent of the hottest SLN in the bed. Pathological handling of the SLNs was as follows: (1) SLNs 3 to 4 mm in maximum dimension were entirely embedded, (2) larger lymph nodes up to 1.0 cm were bisected parallel to the long axis and both halves entirely submitted cut face down in cassettes, and (3) SLNs larger than 1.0 cm were transected into three, four, or more pieces at 2-3 mm intervals and all sections were embedded. One hematoxylin and eosin-stained section and two immunostains (S100 and HMB-45) of each SLN were cut as serial sections 1, 2, 3, and 4, respectively, after obtaining a full-face section.

### 2.3. Statistical Analysis

 Age was categorized according to the decade and as a continuous variable.

Both age categorization schemes were used in univariate and multivariable models to examine primary tumor characteristics, recurrence patterns, and survival. For univariate analysis a Chi-Square was used for categorical variables and an ANOVA test used for continuous variables. For multivariable analysis, a binomial logistic regression was used. Survival and time to recurrence were assessed with Kaplan Meier curves and log rank computation. A *P* value of less than 0.05 on two-tailed analysis was used as the metric for statistical significance.

## 3. Results

 Complete data was available on 1244 SLN biopsies, performed between 1994 and 2009. 1019 (81.9%) were SLN negative, 742 (59.6%) were male, 280 (22.5%) were ulcerated, the mean Breslow depth was 2.45 mm, and distribution by site was 194 (15.6%) head and neck, 281 (22.6%) upper extremity, 501 (40.3%) trunk, and 267 (21.5%) lower extremity. Each demographic variable stratified by decade is found in Table 1. With 80.3 mean months of followup, 235 patients recurred: 104 distant, 50 regionally, 22 in transit (without regional basin involvement), and 22 local; 37 recurrences were combined sites.

**Figure 1:**
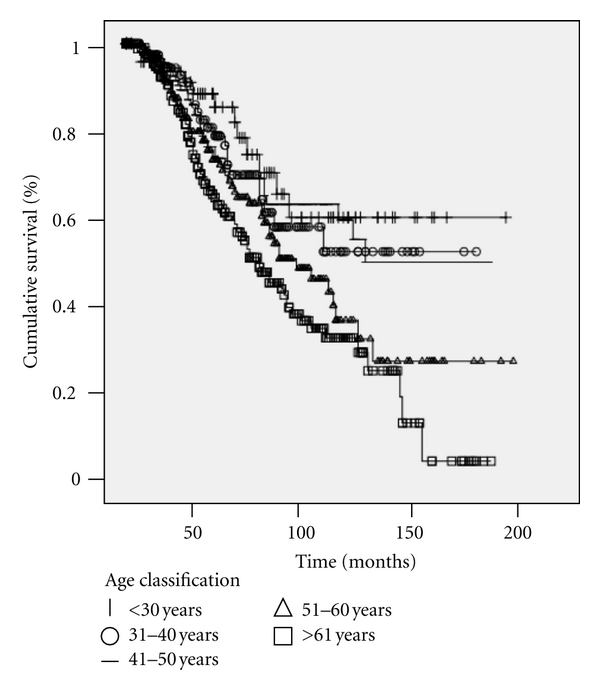
Increased age is associated with poorer survival (*P* = 0.01).

Incorporating the same previously described clinicopathologic characteristics, a multivariable model examining overall melanoma specific survival was used, using age as both a continuous variable and as a categorical variable by decade, demonstrating that age is associated with worse survival in both paradigms (Tables 2(a) and 2 (b)). Using Kaplan Meier survival curves as a univariate representation of survival, we further demonstrate that increased age (by decade) is associated with worse prognosis (*P* = 0.01) (Figure 1). When stratifying by decade on Kaplan Meier analysis, all decades demonstrated that head and neck primary site was a poor prognostic sign (≤30 yrs, *P* = 0.02; 31–40 yrs, *P* = 0.001; 41–50 yrs, *P* < 0.001; 51–60 yrs, *P* = 0.001; ≥61 yrs, *P* = 0.002 (plots not shown)).

We compared both regional and distant recurrence patterns stratified by age. SLN-negative patients (and not SLN-positive patients) were analyzed affording a sample size of 107 (8.6%). We found a nonstatistically supported trend of increased distant recurrences by age after a negative-SLN biopsy (*P* = 0.13) (Table 3). Using the same described multivariable model (with age categorized by decade), our data demonstrate that increased age is associated with increased risk of distant recurrence over regional recurrence (Table 4). A similar trend is evident inversely, as increased age is associated with a trend toward decreased SLN positivity on multivariable analysis (Table 5).

## 4. Discussion

 As the application of SLN biopsy in melanoma becomes more widespread, it is not surprising that there is a growing body of the literature of retrospective studies examining clinicopathologic variables and recurrence patterns in melanoma after SLN biopsy [1, 5]. These retrospective studies, like ours, are invaluable in that they help to characterize the questions that we should ask and they tailor our thinking about the biology of the disease. However, with this increasing body of literature there are expected controversies. The limitations of retrospective analyses generate an inherent ambiguity in the significance of the data. Our study addresses one of those such growing paradoxes in the SLN literature in melanoma. Increasing age has been associated with a lower frequency of SLN positives despite both increasing age and SLN positivity being poor prognostic features [5, 7–9].

 Increased age is associated with poor prognosis in melanoma [2, 3, 10, 11]. Multiple reports have suggested that this finding is both an independent association and secondarily related to correlations with other well-known poor prognostic features. Chao et al. in the Sunbelt Melanoma Group, looking at 3076 patients, showed that age was associated with increased Breslow depth, the incidence of ulceration and regression, and the proportion of male patients [5]. Our data support their findings, (however we did not assess regression in our analysis). Further, they uniquely concluded that increasing age was independently associated with more SLN negatives on multivariable analysis. This study was pivotal in that it was the first to suggest that there may be age-related differences in recurrence based on the paradox that increasing age is associated with more distant recurrences despite having more SLN-negative biopsies. However, their followup was only 19 months, and they found no difference in regional versus distant recurrences. Sassen et al. at the Melanoma Institute Australia with a sample size of 2303 reached a similar conclusion and that there was no difference in distant versus regional recurrence based on age [11].

Younger age is independently associated with more positive-SLN biopsies [5, 12, 13]. This phenomenon has led some groups to suggest that younger patients be given a lower threshold for SLN biopsy than their older counterparts [6]. Potential biologic explanations for this epidemiologic finding are that younger patients have “more competent immune systems,” or that lymphatic function may be impaired in older patients [5, 14, 15]. Unfortunately, the intricacies of these hypotheses have not been mechanistically or empirically described. The Melanoma Institute Australia attempted to address a mechanism for this finding. They hypothesized that younger patients, despite their high frequency of SLN positives, harbor fewer metastatic nodes because of a more intact immune system. But on examination of all positive nodes (SLN and non-SLN), the mean total number of positive nodes removed did not vary according to age [11]. Examining the same issue, Conway et al. at the John Wayne Cancer Institute were successful at demonstrating age-related lymphatic dysfunction specifically for melanoma patients. By examining mean radioactivity counts in axillary, inguinal, and cervical lymph node basins, there was a statistically significant trend with lower counts in all basins in older patients. They proposed that increasing age can modify metastatic patterns [16]. To date, this is the only study to address anatomic and protective immunity differences as they relate to age and melanoma. Potentially the mitotic index, a pathologic feature with established prognostic value, may act as a surrogate marker for biologic activity and recurrence. We have recently begun to analyze this factor in our database, and cannot comment on its utility in our hypothesis.

 Our study, with an extended mean followup (80.3 months) is the first to suggest that increased age is associated with increased distant metastasis, and this may explain the worse prognosis for these older patients. But we also conclude, like the Sunbelt Group and the Michigan cohort, that younger patients are independently associated with fewer positive SLNs and better survival. This conclusion in our dataset was upheld on multivariable analysis, albeit not as strong as other prognostic features (Table 5). The only fundamental differences in our study from the Sunbelt Trial and the Australia group as mentioned previously were that our group's SLN biopsies were performed by only 3 surgeons (KAD, GWC, DRM) between 1998 and 2009, as opposed to multiple centers with many surgeons. Further, we had a larger proportion of head and neck cases 15.6%, a feature showing to have increasing prognostic value and unique SLN drainage implications [17].

 While studies like ours and others continue to pose new questions and conundrums, the future of retrospective probabilistic models which truly elucidate the benefits and prognostic value of SLN lies in elegant predictive nomograms that take into account more specific features of melanoma and age-related variations in primary tumor characteristics and host immunity. The features within these prediction models will incorporate a mechanistic foundation of SLN metastasis which we currently have not uncovered.

## Figures and Tables

**Table 1:** Patient clinicopathologic variables, based on decade of life.

Variable	All patients	≤30 yrs	31–40 yrs	41–50 yrs	51–60 yrs	≥61 yrs
*P* values calculated for deciles		*n* = 110	*n* = 173	*n* = 268	*n* = 299	*n* = 394
SLN						
SLN−	1019 (81.9)	86 (78.2)	136 (78.6)	221 (82.5)	242 (80.9)	334 (84.8)
SLN+	225 (18.1)	24 (21.8)	37 (21.4)	47 (17.5)	57 (19.1)	60 (15.2)
*P* = 0.32						

Gender						
Male	742 (59.6)	54 (49.1)	86 (49.7)	143 (53.4)	199 (66.6)	260 (66.0)
Female	502 (40.4)	56 (50.9)	87 (50.3)	125 (46.6)	100 (33.4)	134 (34.0)
*P* < 0.001						

Ulcer						
Ulcerated	280 (22.5)	20 (18.2)	28 (16.2)	62 (23.1)	66 (22.1)	104 (26.4)
No ulcer	964 (77.5)	90 (81.8)	145 (83.8)	206 (79.6)	233 (77.9)	290 (73.6)
*P* = 0.01						

Breslow depth (mm) (mean)	2.45	2.23	1.82	2.38	2.40	2.86
*P* < 0.001						

General site						
Head & Neck	194 (15.6)	21 (19.1)	16 (19.2)	31 (11.6)	42 (14.0)	84 (21.4)
UE	281 (22.6)	21 (19.1)	32 (18.5)	63 (23.5)	72 (24.1)	93 (21.7)
Trunk	501 (40.3)	47 (19.1)	83 (48.0)	116 (43.3)	124 (41.5)	131 (33.3)
LE	267 (21.5)	21 (42.7)	42 (24.3)	58 (21.6)	61 (20.4)	86 (21.6)
*P* < 0.01						

**Table:** (a)

Variable	Hazard ratio	95% confidence interval	*P* value
SLN POS	4.8	3.35–6.87	<0.001
Male	1.5	1.07–2.20	0.02
Age (continuous)	1.02	1.01–1.03	0.02
Breslow depth (≥1 mm)	2.9	1.15–7.35	0.03
Ulceration	2.24	1.58–3.16	<0.001
Head and neck site	3.07	2.09–4.51	<0.001

**Table:** (b)

Variable	Hazard ratio	95% confidence interval	*P* value
SLN POS	4.78	3.34–6.86	<0.001
Male	1.51	1.05–2.17	0.02
Age			
<30 versus 31–40 yrs	1.56	0.70–3.56	0.28
<30 versus 41–50 yrs	1.36	0.63–2.94	0.44
<30 versus 51–60 yrs	2.07	0.99–4.33	0.05
<30 versus ≥61 yrs	2.68	1.31–5.47	0.01
Breslow depth (≥1 mm)	2.97	1.17–7.52	0.02
Ulceration	2.27	1.61–3.22	<0.001
Head and neck site	3.06	2.07–4.51	<0.001

**Table 3:** Regional versus distant recurrence based on decade of age, (including only SLN NEG, *n* = 107).

Recurrence pattern	≤30 yrs	31–40 yrs	41–50 yrs	51–60 yrs	≥61 yrs	Total
*P* = 0.13	*n* = 110	*n* = 173	*n* = 268	*n* = 299	*n* = 394	
Regional	1 (33.3)	7 (58.3)	7 (36.8)	6 (23.1)	13 (27.7)	34 (31.8)
Distant	2 (66.7)	5 (41.7)	12 (63.2)	20 (76.9)	34 (72.3)	73 (68.2)

Total	3	12	19	26	47	107

**Table 4:** Multivariable model of distant recurrence versus regional recurrences (only SLN negatives and distant/regional recurrences, *n* = 107).

Variable	Hazard ratio	95% confidence interval	*P* value
Male	1.90	0.72–5.00	0.19
Age			
<30 versus 31–40 yrs	2.10	0.83–9.21	0.25
<30 versus 41–50 yrs	3.00	0.68–13.3	0.15
<30 versus 51–60 yrs	4.64	1.05–20.6	0.04
<30 versus ≥61 yrs	2.78	0.70–11.0	0.15
Breslow depth (≥1 mm)	1.90	0.72–5.00	0.19
Ulceration	1.96	0.74–5.24	0.18
Head and neck site	0.83	0.32–2.15	0.71

**Table 5:** Multivariate model for predicting SLN positivity with age based on quartiles demonstrates a trend that increasing age is associated with decreased SLN positivity.

Variable	Hazard ratio	95% confidence interval	*P* value
Male	1.76	1.28–2.42	0.001
Age			
<30 versus 31–40 yrs	1.01	0.56–1.85	0.96
<30 versus 41–50 yrs	0.68	0.39–1.20	0.19
<30 versus 51–60 yrs	0.75	0.43–1.31	0.31
<30 versus ≥61 yrs	0.53	0.30–0.91	0.02
Breslow depth (≥1 mm)	7.38	2.68–20.30	<0.001
Ulceration	2.13	1.54–2.94	<0.001
Head and neck site	0.80	0.53–1.22	0.31
